# Diet, Advanced Maternal Age, and Neonatal Outcomes: Results from the GESTAGE Study

**DOI:** 10.3390/nu17020321

**Published:** 2025-01-17

**Authors:** Maria Puche-Juarez, Juan M. Toledano, Daniel Hinojosa-Nogueira, Catalina de Paco Matallana, Javier Sánchez-Romero, Julio J. Ochoa, Maria Paz Carrillo, Estefanía Martín-Álvarez, Javier Diaz-Castro, Jorge Moreno-Fernandez

**Affiliations:** 1 Department of Physiology, Faculty of Pharmacy, Campus Universitario de Cartuja, University of Granada, 18071 Granada, Spain; mpuchej@ugr.es (M.P.-J.); jjoh@ugr.es (J.J.O.); javierdc@ugr.es (J.D.-C.); jorgemf@ugr.es (J.M.-F.); 2 Institute of Nutrition and Food Technology “José Mataix Verdú”, Biomedical Research Centre, University of Granada, 18016 Armilla, Spain; 3 Nutrition and Food Sciences Ph.D. Program, University of Granada, 18071 Granada, Spain; 4 Biomedicine Research Institute of Málaga, IBIMA, and Nanomedicine Platform, BIONAND, Clinical Management Unit of Endocrinology and Nutrition, Virgen de la Victoria University Hospital, 29010 Málaga, Spain; dhinojosa@ugr.es; 5 Department of Obstetrics and Gynecology, ‘Virgen de la Arrixaca’ University Hospital, 30120 Murcia, Spain; katy.depaco@gmail.com (C.d.P.M.); javier.sanchez14@um.es (J.S.-R.); 6 Department of Obstetrics, Gynecology, Surgery and Pediatrics, University of Murcia, 30120 Murcia, Spain; 7 Institute for Biomedical Research of Murcia, IMIB-Arrixaca, 30120 Murcia, Spain; 8 Instituto de Investigación Biosanitaria (IBS) (E15-EXPODIET, MP19), 18016 Granada, Spain; 9 Department of Obstetrics & Gynaecology, Virgen de las Nieves University Hospital, 18014 Granada, Spain; mpazcb@gmail.com; 10 Unit of Neonatology, Pediatric Service, Hospital Universitario Materno-Infantil Virgen de las Nieves, 18014 Granada, Spain; estefaniamartin82@gmail.com

**Keywords:** pregnant women, pregnancy, advanced maternal age, dietary recommendations, early nutrition, fetal anthropometry, neonatal outcomes, newborn

## Abstract

Maternal nutrition during pregnancy plays a pivotal role in influencing both maternal and fetal health, impacting neonatal anthropometric outcomes and long-term disease susceptibility. An advanced maternal age (AMA ≥ 35 years) has been linked to increased risks of obstetric complications and adverse neonatal outcomes, yet its specific nutritional profile remains underexplored. **Background/Objectives**: This study aimed to evaluate the nutrient and polyphenol intakes of women at an AMA compared to those of a younger control group and to investigate associations with neonatal anthropometric measures. **Methods**: A cohort of 200 pregnant women, stratified into AMA and control groups, completed a food frequency questionnaire during the second trimester. Neonatal anthropometric data were collected at delivery. **Results**: Intakes of fiber, zinc, copper, selenium, vitamins E, B1, B3 and folate were lower in the AMA group in comparison with the control values. Negative correlations were found between fiber, vitamin A and vitamin E and the head circumference of the newborn, with fiber being identified as a potential predictor of this parameter. **Conclusions**: Despite some limitations, such as the fact that the FFQ was completed only once during pregnancy and the cross-sectional design of the study, the findings highlight notable nutritional deficiencies among AMA women, which may influence neonatal outcomes such as head circumference. These results underscore the need for nutritional guidelines and supplementation strategies tailored to pregnant women over 35 years of age.

## 1. Introduction

Nutrition during pregnancy has been studied as a window of opportunity to mitigate and even prevent complications not only during this period [1], like the risk of developing gestational diabetes [2] or labor complications [3], but also in the proper health of the fetus in utero [4] and in subsequent stages of life, such as the occurrence, for example, of certain allergies [5]. This is what Godfrey and Robinson [6], on the basis of Barker’s hypothesis, proposed in 1990, named “nutrition programming”, suggesting that some chronic diseases can be originated from early nutritional status.

There are many factors, according to the developmental origins of health and disease (DOHaD) hypothesis, that can influence both the wellness of the mother and the development of the fetus and that may produce changes later in life, one of which is the age of the mother [7]. The age range of first-time moms has risen significantly in recent years. Many researchers suggest that the increased involvement of women in higher education and the workforce, as well as their aspirations for financial security, are some of the potential causes of this [8,9]. An advanced maternal age (AMA) is defined as ≥35 years at delivery, although some studies refer to it as ≥40 years, associating pregnancy age with adverse neonatal and obstetric outcomes such as hypertension, gestational diabetes, preterm delivery, pre-eclampsia, placenta previa, placental abruption, chromosomal abnormalities, low birth weight, intrauterine fetal growth restriction, macrosomia, a low Apgar score and perinatal mortality [10], which are problems that are more pronounced in women of an extremely advanced maternal age (EMEA) (≥45 years) [11]. According to Eurostat [12], the mean age of pregnancy in Europe is 29.7 years. In Spain, the average is 32 years [13], and the percentage of primiparous mothers over 40 years old is the highest in the European Union [14].

Another factor is the nutritional status of the mother, which can directly affect maternal outcomes, such as weight gain during pregnancy, as well as neonatal outcomes, including fetal development, birth weight and overall health during childhood [15]. Both maternal undernutrition and overnutrition are well-established risk factors for neonatal health [16]. However, even mothers with apparently normal weights and adequate nutrition may lack critical nutrients, potentially leading to alterations in the newborn’s anthropometric measures, which have been linked to health outcomes across infancy, childhood, adolescence and adulthood [17].

In this sense, there is a wide variety of nutrients obtained from the diet that can affect maternal health and fetal development, such as macronutrients, which provide energy to the body, are necessary for both the mother and the fetus for the creation of tissues and their correct development [18] and have been associated with length and weight at birth [19,20]. On the other hand, all micronutrients are equally important in terms of a normal healthy body, but in a concrete physiological status like pregnancy, some of them become even more important, like iron, folate, calcium and vitamin D [21], among others, and it is necessary to supplement many of these during pregnancy, as their deficiency has been linked to fetal outcomes. Finally, polyphenols, secondary metabolites derived from plants [22] and found in vegetables, fruits, grains, nuts, legumes, cocoa and related foods, have beneficial health effects, including during pregnancy [23]. Interestingly, some studies have linked their consumption during pregnancy to certain complications and anthropometric measurements in newborns [23,24,25].

Regarding the amounts of macro- and micronutrients that each population should consume, there are various organizations that define dietary reference values (DRVs) for nutrients. In Spain, these guidelines are produced by the Spanish Food Safety and Nutrition Agency (Agencia Española de Seguridad Alimentaria y Nutrición, AESAN, Madrid, Spain) [26]. It should be noted that in the guidelines from the AESAN for the Spanish population, the DRVs of macro- and micronutrients are divided by populations of different age ranges and also for pregnant and lactating women. Nevertheless, these guidelines do not distinguish between age groups of pregnant women.

Scientific evidence to date reveals dietary habits during pregnancy, helping to shed light on adequate micronutrient intake, which is essential for the proper development of the fetus. However, maternal age, especially in primiparous women, has not been a factor taken into account in the study of nutrition during pregnancy. Therefore, the aim of this study was to assess the consumption of dietary macro- and micronutrients, as well as polyphenols, of pregnant women over 35 years of age compared to those under 35 years of age and to look for possible associations between the dietary habits of these women and the anthropometric measurements of their newborns with the purpose of establish correct dietary recommendations for pregnant women, adjusted to their age, so that they could be included in clinical and nutritional guidelines.

## 2. Materials and Methods

### 2.1. Subjects

A total of 200 pregnant women were recruited. All of them were around 12 weeks of gestation from two hospitals, Hospital Materno-Infantil Virgen de las Nieves (Granada, Spain) and Hospital Universitario Virgen de la Arrixaca (Murcia, Spain). The recruitment period was between June 2022 and January 2024. This population belongs to a multidisciplinary study with the aim of assessing the effect of gestational age (GESTAGE) on different physiological, metabolic and nutritional aspects.

Healthy and nulliparous women with single pregnancy were recruited around the 12th week of gestation in their medical consultation service during one of the first ultrasound examinations. Inclusion criteria were as follows: freely agree to participate in the study and have the informed consent signed by the volunteer or the parents/legal guardians of the newborn; pregnant woman with a normal pregnancy course; body mass index of 18–30 kg/m^2^ at the start of pregnancy and singleton gestation. On the other hand, exclusion criteria were as follows: chronic disease requiring long-term treatment, malnutrition, morbid obesity or underweight, chromosomal or congenital malformations, uterine growth retardation, fetal death, non-acceptance of informed consent to participate in the study by the volunteer or the parents/legal guardians of the newborn. The study has been approved by the Bioethics Committee for the Research with Humans (reference 27/04/2020/4/2020).

After being properly informed about the purpose and nature of the investigation and giving their informed consent, the mothers were placed in one of the study groups: the control group (<35 years old) and the AMA group (≥35 years old). However, during the course of pregnancy, there were losses of 7% in the control group and 9% in the AMA group. The final study groups at the time of delivery were as follows: control group, *n* = 93; AMA group, *n* = 91. Routine clinical analytical parameters were monitored during the delivery, and at the same time, the samples required for further analysis were collected. Participants were free to withdraw from the study at any time in accordance with the Declaration of Helsinki.

### 2.2. Data Collection

Each hospital provided data for each delivery, such as date of delivery, week of gestation, day of gestation, start of labor (spontaneous, induced, elective cesarean section), indication for induced labor, end of labor (spontaneous, vaginal surgery, cesarean section), cm of dilatation at which labor monitoring starts, duration of labor in minutes, amniotic fluid characteristics, term of labor from 5 cm, umbilical cord circling, resuscitation. Furthermore, anthropometric data of the newborn were also provided by each hospital, including weight, length and head circumference. These data were collected by hospital staff according to standardized guidelines and protocols [27,28]. All data were coded and anonymized in compliance with the General Data Protection Regulation (GDPR).

### 2.3. Dietary Assessment

A semi-quantitative food frequency questionnaire (FFQ) was used to assess total nutrient intakes for the dietary record, which had previously been validated in the Spanish pregnant women population [29]. In brief, the FFQ included a variety of foods (87 items), classified into seven groups such as dairy products; eggs, meats and fishes; vegetables and legumes; fruits; bread, cereals and others; oils, fat and processed products; drinks and miscellaneous. Furthermore, the portion size of each food item was specified according to the food portion atlas ENALIA [30]. The FFQ comprises 9 possible responses based on the frequency of consumption, as follows: never; 1–3 times/month; 1/week; 2–4/week; 5–6/week; 1/day; 2–3/day; 4–5/day; 6 or more/day. The FFQ was completed once during pregnancy, in the period between weeks 15 and 20 of gestation. To reduce bias and improve the response rate and accuracy of the data obtained, the FFQs were administered by trained personnel, a nutritionist, who helped to answer the questions. The frequency data were converted into grams of food, and the grams of food were converted into nutrients. The Spanish food database Novartis [31] was used to estimate the intake of different nutrients like total energy, macronutrients (total protein, total fat and carbohydrates) and micronutrients (main dietary minerals and vitamins).

### 2.4. Estimation of the Total Intake of Polyphenols

Polyphenol intakes were estimated using FFQ data and the Phenol-Explorer 3.6 database [22]. This resource provided data on the total polyphenol content of foods using the Folin–Ciocalteu method and the concentrations of individual polyphenol families and subfamilies [22,32]. The total amounts of each polyphenol subfamily were used to estimate the total polyphenol intake of each participant. The subfamilies of phenolic compounds were categorized into several main groups according to the Phenol-Explorer criteria: (a) flavonoids (including anthocyanins, chalcones, dihydrochalcones, dihydroflavonols, flavanols, flavanones, flavones, flavonols and isoflavonoids); (b) phenolic acids (such as hydroxybenzoic acids, hydroxycinnamic acids, hydroxyphenylacetic acids, hydroxiphenylpropenes acids and hydroxyphenylpropanoic acids); (c) lignans (lignans); (d) stilbenes (stilbenes); and (e) other polyphenols (alkylmethoxyphenols, alkylphenols, hydroxybenzaldehydes, hydroxybenzoketones, hydroxycoumarins, methoxyphenols, naphthoquinones, tyrosols and other polyphenols). In order to estimate the polyphenol content of complex foods, including some processed foods, the samples were separated according to their main ingredients, following typical commercial recipes, and adjusted using cooking yield factors [22,32,33].

### 2.5. Comparison with the Dietary Intake References

The average nutrient intakes per group were calculated from the data obtained from the FFQ food consumption estimation. These group averages were then compared with the reference values indicated by the AESAN (Spanish Agency for Food Safety and Nutrition) [26] for the pregnant population. For the mean comparison and the dietary reference values (DRVs) for Spanish pregnant women, the variables were log-transformed in order to represent them on the same scale.

### 2.6. Statistical Analysis

The sample size calculation was carried out in accordance with the specific objectives pursued and on the basis of similar studies published in the scientific literature in which the same type of samples was used [34,35,36]. The following assumptions have been taken into account for this calculation: significance level of 5%, power of 80%, tests for unilateral differences and experimental groups balanced in size, also taking into account the loss of approximately 15% for the entire duration of the study. Based on the calculations carried out, a sample size of 100 mothers per group was established, which would ensure sufficient statistical power.

The mean and standard deviation, for continuous variables, and the absolute (n value) and relative (%) frequencies, for categorical variables, were used to characterize the sociodemographic features of the research population.

Before the statistical analysis for the comparison of nutrients and polyphenols intakes between groups, all variables were examined for homogeneous variance and normality using the Levene and Kolmogorov–Smirnoff tests, respectively. When the data were normally distributed, a *t*-test was applied; if the data were not normally distributed, the U-Mann–Whitney test was used, a non-parametric test for non-paired samples where *p* < 0.05 was considered to indicate statistical significance. GraphPad Prism software version 9.4.1 was used for this statistical analysis [37].

After checking that the data did not follow a normal distribution, Spearman correlation was calculated to check the relationship between the intake of all the nutrients measured by the FFQ and the anthropometric measurements of the newborn. All of the correlations with a *p*-value of <0.05 were considered statistically significant, and therefore, data were normalized by z-score in a stepwise model selection. The open-source software RStudio version 4.3.1 was used for this analysis.

## 3. Results

### 3.1. Characteristics of the Study Population

With regard to the anthropometric measurements of the mothers, as well as the method of conception and the final method of delivery, no statistically significant differences were found, as shown in Table 1.

Concerning the anthropometric measurements of newborns at the time of birth, and the gestational age as well as the sex of the newborn, no statistically significant differences were found (Table 2).

### 3.2. Food Group Intake

**Figure 1:**
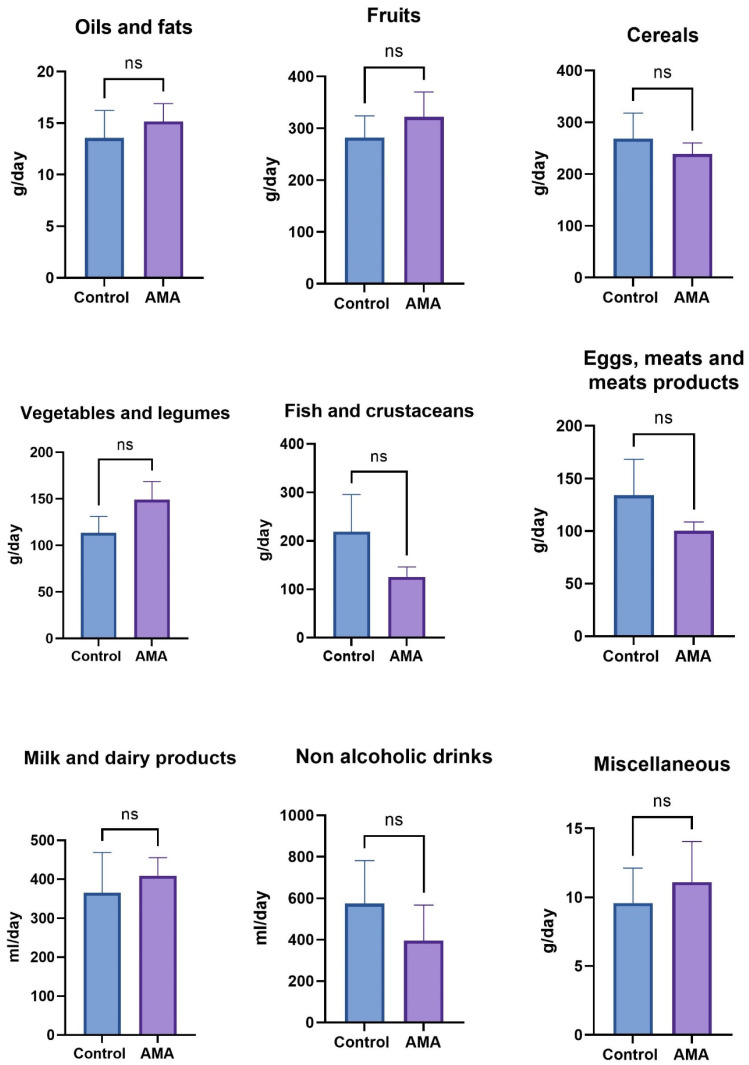
AMA: advanced maternal age; ns: not significant. Comparison between control and AMA at groups of food level, assessed by a food frequency questionnaire.

No significant differences were found in the consumption of foods in the following categories: oils and fats; fruits; cereals; vegetables and legumes; fish and crustaceans; eggs, meats and meat products; milk and dairy products; non-alcoholic drinks and miscellaneous, all of them illustrated in Figure 1.

### 3.3. Nutrient Intake

**Figure 2:**
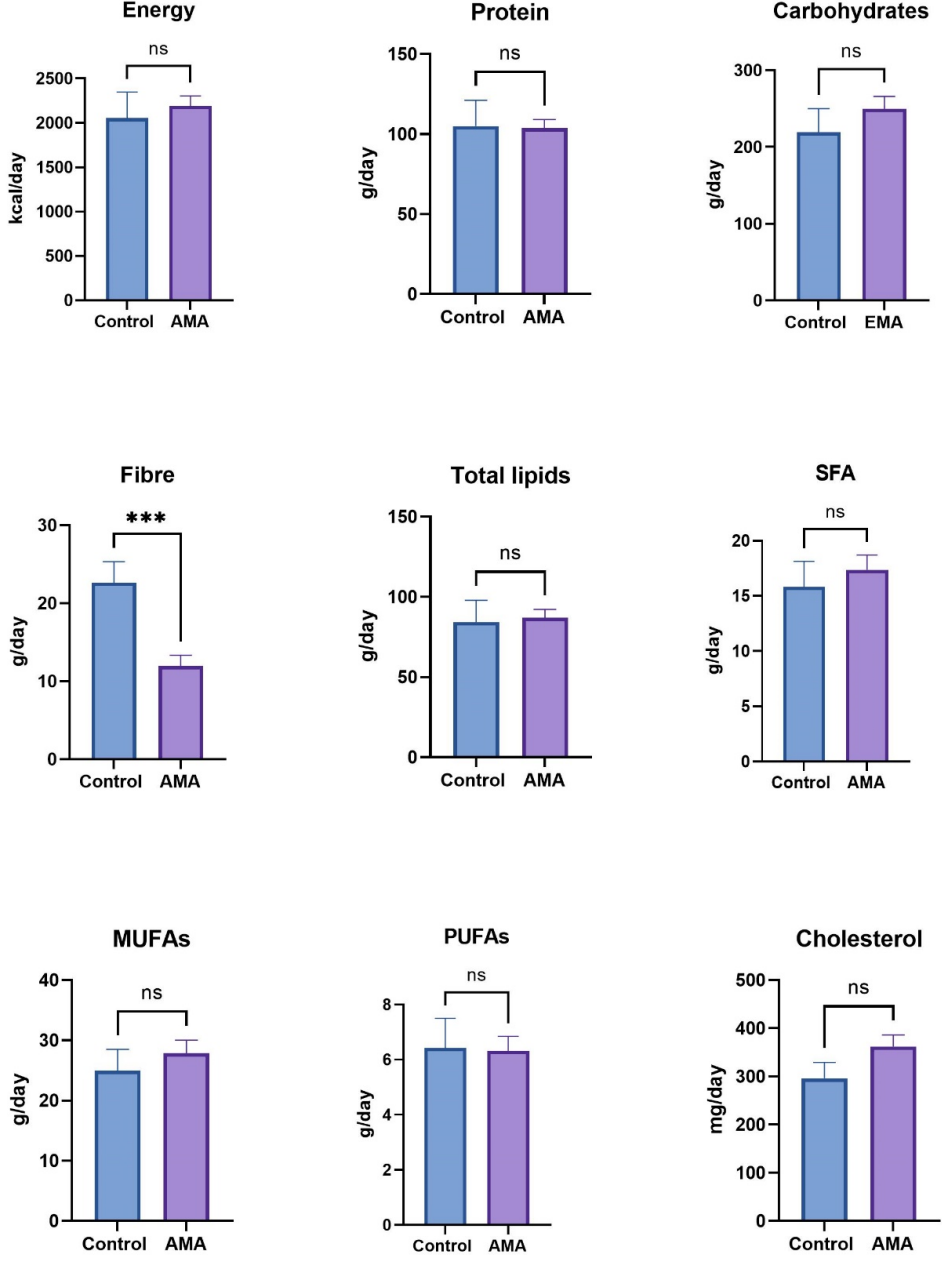
AMA: advanced maternal age; ns: not significant. Comparison between groups of the mean intake macronutrients, evaluated by *t*-test or U-Mann–Whitney (*** *p* < 0.001).

No statistically significant differences were found in terms of total energy, as well as in the total macronutrients. Likewise, when differentiating by type of fat, no statistically significant differences were shown. Fiber consumption, however, was significantly lower in comparison with the control group (22.65 ± 2.6 g/day for the control group and 11.95 ± 1.38 g/day for the AMA group; *p* < 0.05). Differences in the intake of macronutrients, as well as type of fat and fiber, are shown in Figure 2.

The mean intake of minerals and vitamins in both groups is shown in Table 3. A higher intake in the control group was observed compared to the AMA group, with statistically significant differences (*p* < 0.05), for the following minerals: zinc, copper and selenium, and also for the following vitamins: vitamin C, vitamin B1, vitamin B3 and folate.

### 3.4. Polyphenol Intake

Mean intakes of total, groups and subgroups of polyphenols are shown in Table S1 (Supplementary Material). Regarding their intake, no remarkable differences were observed between study groups. The main differences were observed in some components of the flavonoid group: a higher intake was observed in the AMA group compared to the control group in anthocyanins (90.46 ± 9.38 mg/day vs. 56.11 ± 7.103 mg/day, respectively) (*p* < 0.01), flavones (43.06 ± 4.97 mg/day vs. 29.2 ± 3.77 mg/day, respectively) (*p* < 0.05) and flavonols (94.5 ± 10.67 mg/day vs. 58.85 ± 6.62 mg/day, respectively) (*p* < 0.05). A higher intake of hydroxyphenylpropenes was also observed in the AMA group (0.499 ± 0.101 mg/day) compared to the control group (0.197 ± 0.064 mg/day), with statistically significant differences (*p* < 0.05). The rest of the polyphenols studied did not show statistically significant differences.

### 3.5. Comparison with the Dietary Reference Values

**Figure 3:**
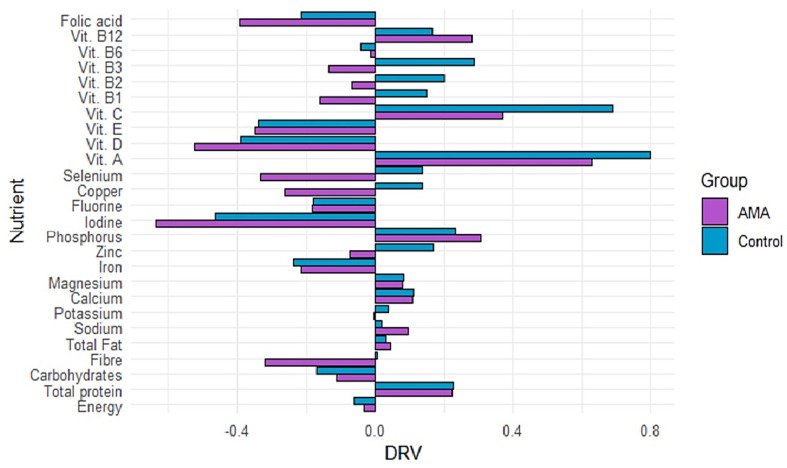
AMA: advanced maternal age; DRV: dietary reference value. Comparison of the mean intake of nutrients by group, with the dietary reference values (DRVs) from the AESAN.

Figure 3 shows a comparison between the intake of nutrients estimated by the FFQ and the DRVs indicated by the AESAN. For most of the nutrients, it is observable that the AMA group is far below the recommendations for minerals like zinc, copper and selenium and vitamins from complex B such as B1, B2 and B3. There are other nutrients like carbohydrates, iron, iodine, fluorine, vitamin D, vitamin E and folic acid which are below the DRVs in both groups.

### 3.6. Correlation Between Nutrient Intake and Anthropometric Measures of the Newborns

The correlation coefficients and *p*-values of the correlations between the nutrient intake during pregnancy and the anthropometric characteristics of the newborn, measured on the labor day are shown in Supplementary Material Table S2.

#### 3.6.1. Anthropometric Association with Macronutrients and Total Energy

**Figure 4:**
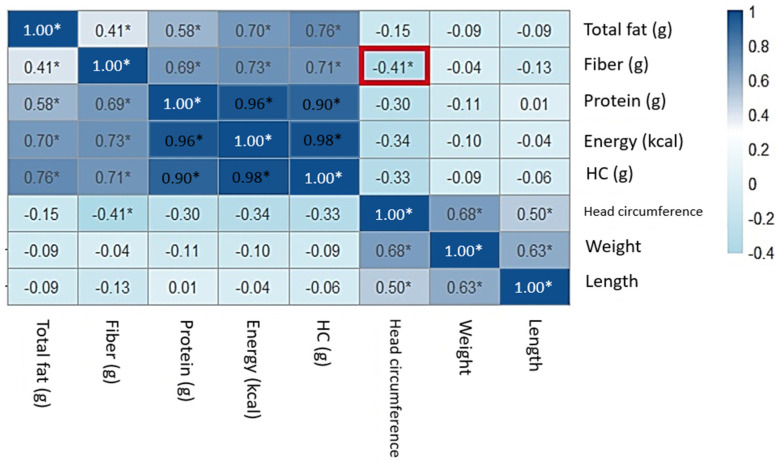
Correlations between energy and macronutrients, including fiber, and anthropometric measures of the newborn (* *p* < 0.05, in red square).

The correlation between anthropometric measures of the neonate and the estimated intake of macronutrients was measured (Table S2), and a heatmap with the correlation coefficients is shown in Figure 4. A significant negative correlation was found between total fiber consumption and the head circumference of the newborn with a coefficient of correlation (r) of −0.41 and *p* = 0.03.

#### 3.6.2. Anthropometric Association with Minerals

As no statistically significant differences were found in the correlation between head circumference, length and weight and the intake of minerals, all data can be observed in Supplementary Material Table S2.

#### 3.6.3. Anthropometric Association with Vitamins

**Figure 5:**
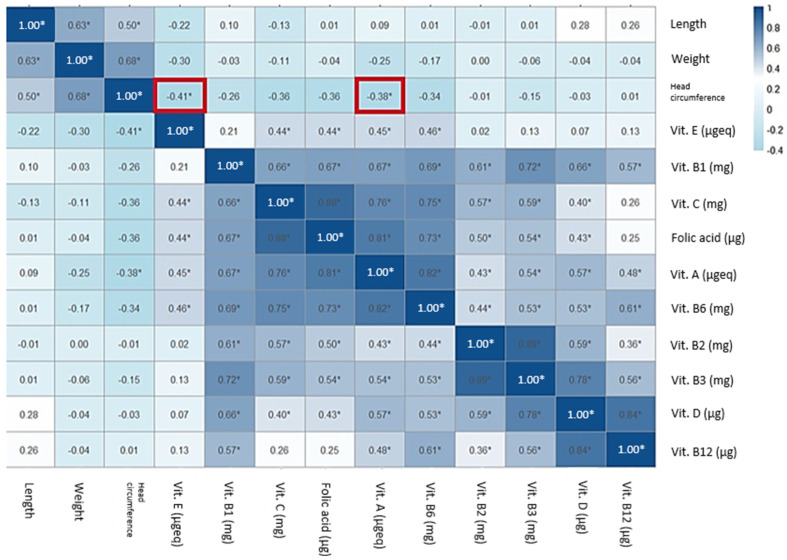
Correlations between vitamins and anthropometric measures of the newborn (* *p* < 0.05, in red square).

With regard to these micronutrients, some negative correlations were found. The heatmap for vitamins can be observed in Figure 5. Significant correlations were found between head circumference, vitamin A (r = −0.38, *p* = 0.01) and vitamin E (r = −0.41, *p* = 0.03).

### 3.7. Regression Model for Associations Between Nutrient Intake and Anthropometric Measures of the Newborns

**Figure 6:**
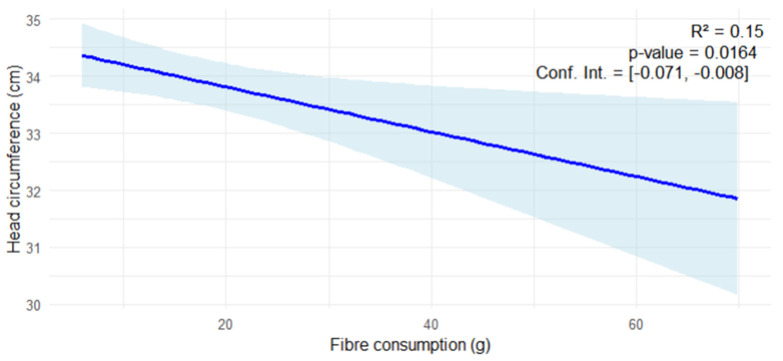
Regression model and linear regression found between fiber and head circumference.

The nutrients that were significantly correlated (*p* < 0.05) with any of the anthropometric measures of the newborn were transformed in a multivariable stepwise regression model as candidate predictors of any of the measures. As shown in Figure 6, consumption of fiber during gestation could be a predictor factor for head circumference with an estimation rate of −0.037.

## 4. Discussion

The present study, to our knowledge, is the first one revealing that pregnant women in general, with no stratification by age, have similar dietary patterns; however, if we deepen our analysis of the diet and analyze each nutrient and dietary pattern, we find certain significant differences in the consumption of several nutrients when separating pregnant women in age groups, according to what the scientific literature considers advanced maternal Age. This study was carried out by estimating nutrient intake using an FFQ validated for the pregnant population [38]. Previous studies have investigated nutrient intake in pregnant women and its influence on fetal outcomes [39,40]; nevertheless, dietary habits and how they can influence maternal and newborn health have never have been studied for AMA and nulliparous pregnancies.

There were no statistically significant differences between AMA and control mothers in the food groups. In the case of this study, both AMA and control pregnant women followed a similar dietary pattern, which could be explained because both cities studied are Mediterranean and both groups followed the typical pattern of a Mediterranean Diet, characterized by being rich in the intake of vegetables, whole grains, nuts, legumes, fish and the use of olive oil as a source of fat [41]. In regard to all of these groups of food, some of them (fruits and cereals, and even starch) play a significant role in modulating glycemic responses [42], which may have effects on some health indicators of the neonate, such as anthropometric measures, which will be discussed below.

With respect to total energy intake, although a slightly higher consumption of kcal per day was found in the AMA group, no statistically significant differences were observed between groups. When compared with the Spanish DRVs for pregnant women, both groups did not achieve them, especially the control group. This result is consistent with other studies revealing that during the first trimester of gestation, pregnant women achieved the recommendation for energy intake, but caloric intake was not increased over the trimesters; therefore, the recommended energy requirements were not met [40,43]. This can be explained because of the transitory increase during the last weeks of the first trimester of pregnancy [44]. In our case, the food questionnaires were answered in the second trimester of pregnancy, where caloric intake is supposed to be higher than in the first trimester, and the DRVs were not reached in both groups. A recent study [40] explained that this is because of mothers’ concern about excessive weight gain; therefore, they limit the kcals in their diet. However, this seems to be a worldwide trend because, as a recently published systematic review concluded, the caloric intake of pregnant women, estimated by FFQs in several studies in different countries, is below their daily intake requirements [45].

Correct macronutrient intake during pregnancy is necessary for the formation of new tissues such as the placenta and fetus and, therefore, for the correct development of the neonate [18].

In the present study, carbohydrates are the only macronutrients that did not achieve the DRVs in both groups. These results are in agreement with previous studies also carried out on Spanish pregnant women [46,47]; however, the average carbohydrate intake is within the recommended levels for this population [45]. When separating by age group, we can observe a slightly but not statistically significantly higher consumption of carbohydrates by the AMA group. Although the daily intake recommendations were not met, we may think that if this group consumes more, they could have better dietary patterns; nevertheless, carbohydrates can be obtained from a number of different sources given different glycemic index (GI) values, which indicate different glycemic responses induced by carbohydrates [48]. Thus, analyzing the food group consumption, we can observe that, although without statistically significant differences, the control group consumes slightly higher amounts of cereals and vegetables than the AMA group. This fact may explain the significant increase in fiber consumption in pregnant women in the control group compared to those in the AMA group, despite the fact that the AMA group consumed more fruits, although this result was not statistically significant. In addition, the control group achieved the correct DRVs for this nutrient. Dietary fiber is a compound of plant-based carbohydrates resistant to digestion by the gastrointestinal tract and can be divided into two groups, soluble fiber (fruits, vegetables and legumes) and insoluble fiber (nuts and cereals), and is also another indicator of the Mediterranean diet [41]. There is another kind of fiber, known as resistant starch, which comes from cooked potatoes and rice [49]. Pregnancy may benefit from diets that are low in GI and rich in fiber, as they help lower blood cholesterol, regulate blood sugar, and encourage laxation [50], and so, this carbohydrate intake pattern may help to achieve a healthy weight during pregnancy and for the neonates [51]. Regard this nutrient, a negative correlation was found between fiber intake and head circumference, indicating that a lower consumption of fiber during pregnancy increases the risk of a bigger head circumference, which is tightly correlated with intracranial volume and is a predictor of cerebral volume. In addition, it has been found that fiber intake during pregnancy may be a predictor of head circumference, with an estimate of −0.037. This means that in our population of Spanish pregnant women, when one unit of fiber intake is added, head circumference is decreased by 0.037 cm. Although this is a fairly low estimate, it is highly relevant, since if it is extrapolated to the general population, it could be said that with 95% probability, this estimate, i.e., the cm that the head circumference will decrease for each unit increase in fiber, will be between −0.07 and −0.008 cm (with these values being the confidence interval of a straight line). Any alteration in the neonatal cephalic size at the moment of birth can indicate varying degrees of neurological dysfunction in childhood [52]. This fact can be explained by several mechanisms. Firstly, as previously explained, the GI and therefore the reduced glucose peaks that fiber consumption induces in the mother’s metabolism, in turn, produce a decrease in insulin secretion. As the fiber intake in the AMA group is statistically significantly lower than in the control group, this mechanism could be altered and as a consequence cause hyperinsulinemia which, in pregnant women, is associated with increased fetal growth, including a larger head circumference [53]. Finally, fiber intake promotes a healthy gut microbiota, which could ferment fiber and produce short chain fatty acids (SCFAs) [54]. These SCFAs, like butyrate, influence insulin sensitivity [55], according to the first mechanism exposed. Although the relationship between fiber intake and head circumference is not fully established and more research is necessary, the results presented in the current study are consistent with the results obtained by Paknahad et al. in 2019 [15]. Not only has this relationship been found, but also, as indicated above, fiber intake in the AMA group is significantly lower than in the control group. Fiber intake decreases across the lifespan [56], and considering all the problems this can induce during pregnancy, for the maternal–fetal health, and the complication that can be associated with those complications, like childhood obesity [57], further research is needed in this field in addition to the revision of the current nutritional guidelines for the pregnant population.

In this study, we found a higher intake of protein compared with previous studies [58], and in both groups, daily consumption was similar. However, it is important to take into account not only the quantity of protein, but also the quality. It is noteworthy that animal protein is considered a complete protein, as it provides the nine indispensable amino acids, while protein from plant-based foods (legumes and nuts) is considered “incomplete” because it often lacks one or more essential amino acids or has insufficient proportions of them [59]. In addition, it is also important to note that the bioavailability of plant-based protein may be lower due to the presence of compounds such as phytates and tannins, which can interfere with the absorption of certain amino acids [60]. Accordingly, when analyzing the food group consumption, the control group presents a statistical trend of consuming more animal protein, while the AMA women consume more vegetable protein, with the potential nutritional consequences that this may have. It is also noticed in both the control and AMA groups that this macronutrient achieves, and even exceeds, the DRVs. As proteins are involved in structural and functional roles, an adequate intake of them will ensure the achievement of the fetal demands [61], but in this case, the intake is a little higher in comparison with the DRVs, and a high protein intake has been linked with an increase in birth weight [62] but with modest effects, which were not observed in our study, as all newborns were within their corresponding percentile. Even so, it has become clear that between 10 and 25% of total energy intake from proteins appears to be safe for pregnant women [50], and in the current study, the intake of both groups was around 20% of total energy. Nevertheless, the quality of those proteins should be considered more than the quantity.

The last macronutrient was total lipids. In both groups, the intake of this macronutrient is slightly elevated, but at normal and healthy rates, both groups meet the DRVs. Other studies have found an intake of 25–35% of the total energy obtained from this macronutrient [63], as in the current study, which is considered an adequate intake. As with the source of origin in protein consumption, it is important to know the origin of the lipids consumed. In our study, although without statistically significant differences, we observed a small tendency towards a higher consumption of cholesterol and saturated fatty acids (SFAs), as well as monounsaturated fatty acids (MUFAs), in the group of mothers ≥35 years old. These fatty acids are necessary for tissue growth and development, especially polyunsaturated fatty acids (PUFAs) like DHA and EPA, which are crucial during pregnancy to satisfy the needs of both the growing fetus and the mother [64]. The correct intake of total lipids as well as different fatty acids has an important role in newborn health since an excessive consumption of fatty acids has been correlated with child obesity, insulin resistance and cardiovascular diseases [65], and this aspect, for the reasons indicated above, should be studied in more depth in the group of mothers of advanced maternal age.

Concerning micronutrients, we found three minerals, zinc, copper and selenium, whose intake was significantly lower in the AMA group and, in addition, was found, in this group, well below the DRVs. Zinc is a component of more than 200 enzymes and of several nucleotides, proteins and hormones and plays a very important role in multiple physiological processes [66]. Zinc deficits contribute to millions of maternal and child deaths annually, mostly in developing countries [67]. They have also been associated with prolonged labor, preterm neonates, fetal growth restriction and low birth weight [68]. Copper and selenium are the next ones whose intake in the AMA group is considerably lower compared to the control group, and their consumption does not reach DRVs in older mothers. Copper is an essential cofactor of enzymes such as cytochrome c oxidase, crucial for brain and neurological development, the deficiency of which can lead to incomplete development not only of brain structures but also of the brain [69], as well as bone structures and connective tissues by being vital in the function of lysyl oxidase, an enzyme involved in the formation of collagen and elastin [70]. On the other hand, selenium plays an important role in fetal development due to its antioxidant function, as it is an essential component of selenoproteins, such as glutathione peroxidase [71]. In addition, oxidative stress from low selenium levels may be related to fetal growth by restricting placental blood flow [72]. Therefore, lower intakes of these minerals may play a crucial role during pregnancy and in neonatal development.

Regarding the vitamin group, vitamin B1 (thiamine), vitamin B3 (niacin), vitamin C and folate consumption are statistically different between groups, being higher in the control group than in the AMA group. Moreover, with respect to the DRVs, vitamins B1, B2 and B3 are below them. In this sense, several studies have linked vitamin B1 deficiency to problems in fetal brain development, due to alterations in the synthesis of lipids and nucleotides in the brain caused by alterations in thiamine-dependent enzyme systems [73]. This deficiency is in contrast with other studies carried out in Spain, reporting similar consumption in pregnant women [40]. In case of folic acid, a water-soluble vitamin of the B group, deficiency causes the accumulation of homocysteine which can lead to adverse pregnancy outcomes and neural tube growth problems. Folate concentrations tend to decrease during pregnancy, caused by fetal demand for folate together with a decrease in folate levels with age, due to factors already mentioned such as changes in the gastrointestinal system and the ability to absorb vitamins and minerals [74]. Therefore, both peri- and post-conception supplementation are essential [75], but despite this, as evidenced in this study, supplementation may not meet the requirements, so it is important to highlight the need to increase the consumption of folate-rich foods, which may help to meet the requirements, along with supplementation, to prevent potential health problems in the newborn. With vitamin C, the pattern is just the opposite compared to the vitamins described above, and although both groups reached the DRVs, the consumption of the control group was significantly higher compared with the AMA group. Vitamin C is an antioxidant vitamin whose main role is the prevention of the onset of oxidative stress [76], which is thought to be a class mechanism in the pathophysiology of certain complications in pregnancy, including intrauterine growth restriction (IUGR), and it has been linked to non-communicable diseases in childhood such as obesity and type 2 diabetes [77]. On the contrary, and based on this mechanism, excessive intake may deregulate this necessary oxidative stress and reduce it excessively, limiting optimal brain growth and thus head circumference. The scientific evidence in favor of supplementation with this vitamin is therefore mixed, and adverse effects have even been observed [76]. Another negative association between head circumference and vitamin A can also be observed. But in this case, both groups reach the proper intake and even exceed it. So, vitamin A is essential for fetal development, as it regulates process like cellular differentiation and organ development. Nevertheless, an excess of vitamin A could have teratogenic effects, interfering with morphologic processes [78], leading to a decrease in head circumference.

Other micronutrients are below the Spanish DRVs for the pregnant population in both study groups; however, their consumption is similar in both groups. Those are iron, iodine, fluorine, vitamin B6 and vitamin D. Iron, essential for producing myoglobin and hemoglobin and supporting cellular processes like growth, respiration and oxygen transport, is required at levels of up to 27 mg/day due to the expansion of maternal red blood cell mass, fetal development and preparation for potential blood loss during childbirth [79]. Similarly, iodine, crucial for regulating metabolism, growth and nervous system development through thyroid hormones, seems to have higher demands due to increased metabolic and hormonal changes, although its effects on fetal anthropometric measures remain under-researched and inconclusive. Vitamin D, primarily synthesized through sun exposure, is entirely supplied by maternal reserves and is vital for fetal development, with deficiencies linked to complications like preeclampsia and small-for-gestational-age newborns, while some studies suggest modest associations with birth weight and bone mass [80,81]. Notably, in the current study, dietary vitamin D intakes are below recommended levels, emphasizing the importance of supplementation. However, it should be noted that many of the micronutrients are frequently supplemented during pregnancy, although this is only advice given by the medical team accompanying the pregnant woman and, like the dietary guidelines, these are not differentiated by age; the doses are recommended equally at any age. Correcting dietary guidelines together with effective supplementation could be recommendable policies for the correct development of the fetus and the prevention of complications during the pregnancy process. Therefore, and for all the reasons discussed above, it would be of great interest to review the current dietary guidelines as well as to include directions on supplementation based on age.

The last compounds evaluated in this study were polyphenols. We have focused on subgroups of polyphenols, and the variability of these between groups of study is quite large. Thus, the intake of the anthocyanin, flavon, flavonol and hydroxiphenilpropene subgroups by diet was shown to be significantly higher in the AMA group than control. The main possible beneficial effects obtained by their intake is related to antioxidant activity, but also, they have been associated with a reduction in cardiovascular diseases [82]. Despite this, an elevated intake of these compounds has been linked to different problems at childbirth such a low birth weight [83,84,85,86,87] based on the associations between polyphenols intake and the constriction of the fetal ductus arteriosus [85]. More important than the intake per se is the fact that flavons and flavonols are part of the flavonoid group, which has been reported to interfere with the absorption of other micronutrients such as iron and folic acid [88], minerals whose intake in this population of mothers in advanced maternal age is well below the dietary recommendations. Studies in pregnant women are scarce and show some controversy between their benefits or their harmful role [89]; even more, the role of polyphenols in pregnancy in AMA has not yet been investigated, so further research is needed in this field.

To the best of our knowledge, this is the first study to evaluate the diet of pregnant women of advanced maternal age. In addition, populations from different Spanish regions were included, thus making the sample more homogeneous and ensuring an adequate sample size for the type of study. However, certain limitations of the present study should be noted. On the one hand, although consumption frequency questionnaires are a validated tool for dietary analysis, they have been self-reported and may be subject to memory bias, underestimation or overestimation. Therefore, trained staff assisted the participants in this study in completing the questionnaire. Another limitation of the study is that dietary intake was only measured at a single point in time, rather than throughout the complete pregnancy period, as well as not taking into account the supplementation, but this study was only focused on dietary food groups. On the other hand, the cross-sectional design of the study means that, despite having observed significant associations between nutrient deficits and some anthropometric measures, the ability to establish casual relationships between them is limited.

The fact that intakes of many micronutrients are much lower in the AMA group without taking into account the supplementation is alarming, as the absorption of these minerals and vitamins is reduced as we grow older. As women age, their ability to absorb minerals and vitamins decreases due to factors such as changes in gastrointestinal function and slower metabolism [90]; this means that pregnancy at an advanced age may start with lower micronutrient stores and face more difficulties in meeting the increased demands of pregnancy due to gastrointestinal changes that make micronutrients less available for the different metabolic reactions in which they are involved and, in the case of pregnancy, in all the processes involved in the correct development of the fetus. This can be translated into the fact that even their supplementation should be reviewed and adapted to their age. This, combined with an increased intake of a group of polyphenols through the diet, appears to be linked to adverse effects on fetal development, making future research in the field of nutrition in advanced maternal age crucial.

## 5. Conclusions

The present study reveals that Spanish pregnant women have micronutrient deficiencies when micronutrients are administered only through their diet, according to the AESAN dietary reference recommendations. These deficits increase even more in women of advanced maternal age, despite having a similar dietary pattern to their control group. In this sense, it has been observed that some micronutrients, especially minerals such as zinc, copper and selenium and vitamins like vitamin C, vitamins B1 and B3 and folate, show intake quite below recommendations in the AMA group, which, as has been described, could have negative consequences for fetal development. This, together with the negative correlation found between fiber intake and head circumference of the newborn, with the group of mothers older than 35 years old being the one in which fiber intake is statistically significantly lower, highlights the importance of considering stratification by age in the design of nutritional guidelines. Current nutritional policies and clinical guidelines must address the unique needs of AMA women by incorporating targeted strategies, including personalized dietary recommendations and supplementation protocols tailored to their specific physiological demands. Such measures could help mitigate the risks associated with nutrient deficiencies and their potential consequences for maternal and neonatal health [91]. Despite the limitations of the study, the results demonstrate the need for longitudinal research to explore causal relationships between maternal diet and perinatal outcomes. An integrated, age-stratified approach could contribute significantly to improving health outcomes for both mothers and newborns.

## Figures and Tables

**Table 1:** Anthropometric measures, method of conception and method of delivery of control group (<35 old) and AMA (≥35 years old).

		Control	AMA
**Weight (kg)**		64.36 ± 5	64.16 ± 13.16
**Height (cm)**		163.55 ± 3.5	163.87 ± 5.89
**BMI (kg/m^2^)**		24.04 ± 2.88	23.84 ± 4.57
**Method of conception**	S (%):	92.5	75.5
	I-OI (%):	1.25	1.01
	IVF-ICSI (%):	5	13.13
	OD (%):	1.25	10.1
**Delivery method**	V (%):	53.12	46.83
	C (%):	31.25	35.44
	VO (%):	15.62	17.72

AMA: advanced maternal age; BMI: body mass index; S: spontaneous; I-IO: insemination–ovulation inducer; IVF-ICSI: in vitro fertilization–intracytoplasmic sperm injection; OD: oocyte donation; V: vaginal; C: cesarean section; VO: vaginal operation. Mean and standard error of the mean are presented.

**Table 2:** Anthropometric measurements, gestational age and sex of newborns of control (<35 years old) and AMA (≥35 years old). Mean and standard error of the mean are presented.

		Control	AMA
**Weight (kg)**		3181.38 ± 47.5	3072.06 ± 512.1
**Length (cm)**		49.8 ± 12.75	49.41 ± 2.54
**Head circumference**		33.42 ± 12.75	34.11 ± 1.66
**Gestational age (weeks)**		38.84 ± 9	38.28 ± 3.21
**Sex**	F (%)	46.03	48.75
	M (%)	53.96	51.25

AMA: advanced maternal age; F: female; M: male. Data are shown as mean ± SEM.

**Table 3:** Mean and standard error of the mean of all the minerals and vitamins estimated by the FFQ in the control and AMA group.

	Control	AMA
**Sodium (mg/day)**	1562 ± 202.5	1862 ± 140.8
**Potassium (mg/day)**	3172 ± 383.8	3473 ± 239
**Calcium (mg/day)**	1086 ± 179.9	96.55
**Magnesium (mg/day)**	315.7 ± 40.6	361.4 ± 24.6
**Iron (mg/day)**	15.69 ± 2	16.5 ± 0.99
**Zinc (mg/day)**	13.35 ± 1.8	8.46 ± 0.62 **
**Phosphorus (mg/day)**	1370 ± 173.4	1623 ± 106.5
**Iodine (µg/day)**	48.37 ± 8.54	46.2 ± 4.48
**Fluorine (µg/day)**	714.2 ± 112.1	709.8 ± 108.6
**Copper (µg/day)**	1388 ± 177.1	653.9 ± 55.1 ***
**Selenium (µg/day)**	64.19 ± 8.1	25.53 ± 2.59 ***
**Vit. A (µeq/day)**	3983 ± 512.8	3001 ± 218.2
**Vit. D (mg/day)**	6.1 ± 0.83	4.47 ± 0.55
**Vit. C (µeq/day)**	293.4 ± 43.34	150.9 ± 14.72 **
**Vit. E (µg/day)**	5.47 ± 0.88	5.39 ± 0.48
**Vit. B1 (mg/day)**	1.53 ± 0.21	0.889 ± 0.072 **
**Vit. B2 (mg/day)**	1.76 ± 0.24	1.2 ± 0.08
**Vit B3 (mg/day)**	21.83 ± 3.5	11.02 ± 0.87 **
**Vit. B6 (mg/day)**	1.722 ± 0.228	1.83 ± 0.114
**Vit. B12 (µg/day)**	3.811 ± 0.525	4.979 ± 0.441
**Folate (µg/day)**	256.4 ± 24.76	201.4 ± 22.85 *

Data are shown as the mean values ± SEM. Significantly different from the control group (* *p* < 0.01, ** *p* < 0.01, *** *p* < 0.001, Student’s *t*-test). AMA: advanced maternal age.

## Data Availability

The original contributions presented in this study are included in the article/Supplementary Materials. Further inquiries can be directed to the corresponding author.

## Supplementary Materials

The following supporting information can be downloaded at https://www.mdpi.com/article/10.3390/nu17020321/s1: Table S1: Mean and standard error of the mean of total polyphenols, groups of polyphenols and subgroups of polyphenol intake; Table S2: Correlations between nutrient intake and anthropometric measures of the newborn.
