# Supplementary material for: Diet, Advanced Maternal Age, and Neonatal Outcomes: Results from the GESTAGE Study
Source: Nutrients. 2025 Jan 17;17(2):321. doi: 10.3390/nu17020321 (PMC11769228; doi:10.3390/nu17020321)
Supplement: Supplementary file 1 [file nutrients-17-00321-s001.zip › nutrients-3396833-supplementary.pdf]

Table S1. Mean and standard error of the mean of total polyphenol, groups of polyphenols and subgroups of polyphenols intake.

Group	Subgroup	Control	AMA
**Polyphenols total**		1509 ± 232	1335 ± 120.9
**Flavonoids**		532.2 ± 92.98	650.4 ± 74.62
	Anthocyanins	56.11 ± 7.103	90.46 ± 9.38 **
	Chalcones	0.0017 ± 0.001	0.000726 ± 0.0004
	Dihydrochalcones	3.185 ± 0.55	2.230 ± 0.42
	Dihydroflavonols	0.193 ± 0.049	0.202 ± 0.031
	Flavanols	352.6 ±58.76	369.1 ± 37.92
	Flavonones	50.69 ± 9.66	36.14 ± 5.53
	Flavones	29.2 ± 3.77	43.06 ± 4.97 *
	Flavonols	58.85 ± 6.62	94.5 ± 10.67 *
	Isoflavonoids	0.473 ± 0.086	0.283 v 0.028
**Phenolic acids**		955.8 ± 202.2	817.8 ± 124.9
	Hydroxibenzoics acids	102 ± 19.18	99.92 ± 13.32
	Hydroxicinnamic acids	879.3 ± 203.8	725 ± 116.7
	Hydroxiphenylacetic acids	3.119 ± 0.818	3.299 ± 0.538
	Hydroxiphenylpropanoic acids	0.223 ± 0.06	0.275 ± 0.05
	Hydroxiphenylpropenes acids	0.197 ± 0.064	0.499 ± 0.101 *
**Lignans**		28.4 ±3.79	35.09 ± 4.129
**Stilbenes**		0.112 ± 0.02	0.136 ± 0.01
**Other polyphenols**		59.01 ± 7.52	64.34 ± 6.26
	Alkylmethoxyphenols	5.94 ± 1.19	4.01 ± 0.49
	Alkylphenols	26.22 ± 4.95	33.53 ± 4.74
	Hydroxybenzaldehydes	0.02 ± 0.004	0.27 ± 0.004
	Hydroxybenzoketones	0.00018 ± 0.00005	0.00037 ± 0.0001
	Hydroxycoumarins	0.0004 ± 0.0001	0.001 ± 0.0004
	Methoxyphenols	0.578 ± 0.15	0.451 ± 0.09
	Naphtoquinones	0.082 ± 0.02	0.03 ± 0.004
	Tyrosols	22.38 ± 4.31	23.8 ± 2.57
	Other polyphenols	5.12 ± 1.36	2.95 ± 0.59

AMA: advanced maternal age. Data are shown as the mean values ± SEM. Significantly different from the control group (* $p < 0.05$; ** $p < 0{,}01$, Student's *t* test).

Table S2. Correlations between nutrients intake and anthropometric measures of the newborn.

	Length (r)	*p*-Value	Weight (r)	*p*-Value	Head Circunference (r)	*p*-Value
**Total energy (kcal)**	-0.04	0.8	-0.1	0.6	-0.34	0.07
**Protein (g/day)**	0.01	0.9	-0.11	0.57	-0.3	0.12
**Carbohydrates (g/day)**	-0.06	0.75	-0.09	0.62	-0.33	0.08
**Total fieber (g/day)**	-0.13	0.48	-0.04	0.85	-0.41*	0.03
**Total lipids (g/day)**	-0.09	0.63	-0.09	0.65	-0.15	0.46
**SFA (g/day)**	0.09	0.64	-0.15	0.44	-0.33	0.09
**MUFA (g/day)**	-0.07	0.72	-0.06	0.75	-0.31	0.1
**PUFA (g/day)**	0.04	0.83	-0.03	0.87	-0.15	0.44
**Cholesterol (mg/day)**	-0.01	0.94	-0.13	0.5	-0.36	0.05
**Sodium (mg/day)**	0.02	0.92	0.08	0.68	-0.16	0.4
**Potasio (mg/day)**	0.05	0.79	0.01	0.95	-0.22	0.25
**Calcium (mg/day)**	0.15	0.43	0.21	0.27	0.06	0.74
**Magnesium (mg/day)**	0.04	0.81	0.06	0.77	-0.14	0.47
**Iron (mg/day)**	0.03	0.89	0.02	0.89	-0.17	0.39
**Zinc (mg/day)**	0.17	0.36	0.21	0.26	-0.12	0.55
**Phosphorus (mg/day)**	0.15	0.4	0.03	0.86	-0.13	0.49
**Iodine (µg/day)**	0.09	0.65	-0.25	0.19	0.27	0.12
**Fluor (µg/day)**	-0.22	0.24	-0.19	0.32	0	0.98
**Copper (µg/day)**	-0.12	0.53	0.13	0.48	-0.28	0.15
**Selenium (µg/day)**	-0.01	0.96	0.09	0.64	-0.18	0.36
**Vit. A (µeq/day)**	0.09	0.62	-0.25	0.18	-0.38*	0.04
**Vit. C (mg/day)**	-0.13	0.49	-0.11	0.57	-036	0.06
**Vit. E (µeq/day)**	-0.22	0.25	-0.3	0.11	-0.41*	0.02
**Vit. D (µg/day)**	0.28	0.14	-0.04	0.83	-0.03	0.86
**Vit. B1 (mg/day)**	0.1	0.61	-0.03	0.86	-0.26	0.18
**Vit. B2 (mg/day)**	-0.01	0.95	0	0.98	-0.01	0.94
**Vit B3 (mg/day)**	0.01	0.95	-0.06	0.77	-0.15	0.44
**Vit. B6 (mg/day)**	0.01	0.95	-0.17	0.37	-0.34	0.08
**Vit. B12 (µg/day)**	0.26	0.17	-0.04	0.83	0.01	0.94
**Folate (µg/day)**	0.01	0.96	-0.04	0.81	-0.36	0.06

Data show coefficients and p-values of the Spearman correlations carried out between the nutrients intake during pregnancy from the mothers, and the anthropometric characteristics of the newborn, measured in the labour day (* $p < 0.05$).
